# Fine mapping of an anthracnose-resistance *locus* in Andean common bean cultivar Amendoim Cavalo

**DOI:** 10.1371/journal.pone.0239763

**Published:** 2020-10-07

**Authors:** Thiago Alexandre Santana Gilio, Oscar P. Hurtado-Gonzales, Maria Celeste Gonçalves-Vidigal, Giseli Valentini, Julio Cesar Ferreira Elias, Qijian Song, Marcial A. Pastor-Corrales

**Affiliations:** 1 Departamento de Agronomia, Universidade Estadual de Maringá, Maringá, Paraná, Brazil; 2 Programa de pós-graduação em genética e melhoramento de plantas, Universidade do Estado de Mato Grosso, Cáceres, Mato Grosso, Brazil; 3 Soybean Genomics and Improvement Laboratory, United States Department of Agriculture, Agricultural Research Service, Beltsville, Maryland, United States of America; Faculty of Agriculture (FoA), Sher-e-Kashmir University of Agricultural Sciences and Technology of Kashmir (SKUAST-K), Wadura Campus, INDIA

## Abstract

Anthracnose, caused by the fungal pathogen *Colletotrichum lindemuthianum*, is one of the world’s most destructive diseases of common bean. The use of resistant cultivars is the most cost-effective strategy to manage this disease; however, durable resistance is difficult to achieve due to the vast virulence diversity of the anthracnose pathogen. Finding new genes with broad-spectrum resistance increases the prospect of designing an effective anthracnose-management strategy. Genetic analysis confirmed the presence of a single, dominant anthracnose-resistance *locus* in AC, which we provisionally named *Co-AC*. Bulk segregant analysis and genetic mapping of two F_2_ populations from the crosses AC × PI207262 and AC × G 2333 were used to determine the position of the *Co-AC locus* in a 631 Kbp genomic region flanked by the SNP markers SS56 and SS92 on the lower arm of chromosome Pv01. By genotyping 77 F_3_ plants from the AC × PI207262 cross using nine additional markers, we fine-mapped the *Co-AC locus* to a significantly smaller genomic region (9.4 Kbp) flanked by the SNP markers SS102 and SS165. This 9.4 Kbp region harbors three predicted genes based on the common bean reference genome, notably including the gene model Phvul.001G244300, which encodes Clathrin heavy chain 1, a protein that supports specific stomatal regulation functions and might play a role in plant defense signaling. Because the *Co-AC* resistance *locus* is linked in *cis*, it can be selected with great efficiency using molecular markers. These results will be very useful for breeding programs aimed at developing bean cultivars with anthracnose resistance using marker-assisted selection. This study revealed the broad-spectrum resistance of AC to *C*. *lindemuthianum* and the existence of the *Co-AC* anthracnose-resistance *locus*. Fine mapping positioned this *locus* in a small genomic region on the lower end of chromosome Pv01 that contained three candidate genes for the *Co-AC locus*.

## Introduction

The common bean (*Phaseolus vulgaris* L.) is by far the most important food legume for human consumption in the world [1,2]. Dry beans are a vital and low-cost source of protein, dietary fiber, complex carbohydrates, and other essential nutrients for millions of people in the world, especially for low-income populations in Africa and Latin America.

Common bean production is impaired by multiple biotic and abiotic constraints [3]. Among more than 80 reported common bean diseases, anthracnose, caused by the hemibiotrophic ascomycete fungus *Colletotrichum lindemuthianum* (Sacc. and Magnus) Briosi and Cavara, is one of the most widespread and recurrent [1–5]. Anthracnose occurs throughout the world, but it is a greater and more significant threat to common bean production in the Americas and Africa, the world’s largest common bean production regions [5–8]. Anthracnose can be devastating, causing severe yield, seed and pod quality losses of up to 95% [9]. Genetic resistance, i.e., planting resistant cultivars, is the most cost-effective and sustainable strategy to control anthracnose in subsistence and commercial agriculture. However, the durability of resistant cultivars is often ended by the appearance of new, virulent strains of *C*. *lindemuthianum* that infect previously resistant cultivars [10,11]. This pathogen is known for its vast virulence diversity, comprising hundreds of virulent races [5,12–15].

Anthracnose resistance in common bean is conferred by multiple single and independent named and mapped genes. Most of these genes have been assigned *Co* symbols, as follows: *Co-1* (with four alleles), *Co-2*, *Co-3* (with four alleles), *Co-4* (with two alleles), *Co-5* (with one allele), *Co-6*, *Co-11*, *Co-12*, *Co-13*, *Co-14*, *Co-15*, *Co-16*, and *Co-17* [6,16–30]. In 2017, a new allele of *Co-1* provisionally named *Co-1*^*HY*^ and a new dominant resistance gene provisionally named *Co-Pa* were reported [8,29]. Several of these anthracnose-resistance genes are located in clusters, where they are tightly linked to other resistance genes (for angular leaf spot, rust, etc.), and these clusters are often positioned at the ends of chromosomes [19,31–36]. Clusters of anthracnose-resistance genes have been reported on Pv01 (*Co-1* and alleles, *Co-14*, *Co-Pa*, *Co-x*, and *Co-w*), Pv02 (*Co-u* and *CoPv02*), Pv03 (*Co-13*, and *Co-17*), Pv04 (*Co-3*, *Co-3*^*2*^, *Co-3*^*3*^, *Co-3*^*4*^*/Phg-3*, *Co-y*, *Co-z* and *Co-RVI*) and Pv07 (*Co-5*, *Co-6*, and *Co-v*). Thus far, all disease resistance genes on chromosome Pv01 are from common bean accessions belonging to the Andean gene pool. These include anthracnose (*Co-1* with five alleles, *Co-1*^*HY*^, *Co-14*, *Co-Pa*, *Co-x*, and *Co-w*), rust (*Ur-9*), and angular leaf spot (*Phg-1*) resistance genes [6,8,16,19,22,29,32].

Until approximately 2006, most anthracnose-resistance genes were found in Mesoamerican cultivars; only the *Co-1* gene and its alleles were found in cultivars from the Andean gene pool [6,16]. Then, beginning in 2008, several additional single, independent anthracnose-resistance genes (*Co-12*, *Co-13*, *Co-14*, *Co-Pa*, *Co-x*, and *Co-w*) that were not reported as alleles of *Co-1* were found in cultivars from the Andean gene pool [18,20,29,32,34,36,37]. Despite this progress in identifying new Andean anthracnose-resistance genes, a need remains to discover additional genes from the underutilized Andean gene pool. Genes of Andean origin often confer effective resistance to races of *C*. *lindemuthianum* that infect Mesoamerican cultivars that are widely grown in Mexico, Central America, and Brazil, as well as in the United States and Canada.

The Nupagri Center of Research Applied to Agriculture (Núcleo de Pesquisa Aplicada à Agricultura) at the State University of Maringa (Universidade Estadual de Maringá), Paraná, Brazil, has collected Andean landraces grown in Brazil for use in crop improvement activities. Several of these landraces have been reported as sources of new anthracnose-resistance genes [18,20,26,29,36,38]. Among these, the Amendoim Cavalo (AC) cultivar, which was collected in the state of Santa Catarina, has a type II growth habit, medium-size seeds (34.15 g per 100 seeds), and the morphological characteristics of the Nueva Granada race of the Andean gene pool [39]. The phaseolin seed protein test as described by Kami et al. [40] showed that the seeds of AC contained phaseolin ‘T’, which is characteristic of common beans from the Andean gene pool [41].

The initial anthracnose-resistance study showed that AC was resistant to the Andean race 2 and the Mesoamerican races 65, 73, and 2047 of *C*. *lindemuthianum* [42]. Races 2, 65 and 73 of *C*. *lindemuthianum* have been reported to occur in multiple states of Brazil, as well as in numerous countries of the Americas, Africa, and other continents [12–15].

Thus, this study showed that AC was resistant to races that were widespread in many countries, suggesting that AC had broad resistance to the anthracnose pathogen. The inheritance of resistance tests conducted by Nanami et al. [42] and during this study indicated that the resistance of AC was conferred by a single dominant gene. Therefore, the objectives of this study were to fine-mapping the anthracnose-resistance *locus* in AC and to develop DNA markers tightly linked to this *locus*.

## Materials and methods

### Population development

AC was crossed with the cultivars PI 207262, G 2333, and Widusa to produce F_1_ and F_2_ plants and F_2:3_ families. The parents, the segregating populations from each of the crosses, and other cultivars used as controls were inoculated with specific races of *C*. *lindemuthianum*. A total of 113 F_2_ plants from the AC × PI 207262 cross were used for initial mapping of the resistance in AC and were inoculated with race 3481 of *C*. *lindemuthianum*. AC was resistant (R), while PI 207262 was susceptible (S). The F_2:3_ families derived from the AC × PI 207262 cross were used for fine mapping and phenotyped for their reactions to race 3481. A second F_2_ population of 189 plants from the AC (R) x G 2333 (S) cross was used to confirm the genetic position of the *Co-AC locus*. A third population was developed to perform a cosegregation analysis using races 23 (Andean) and 3481 (Mesoamerican) of *C*. *lindemuthianum*. The nature of the Amendoim Cavalo resistance was assessed by analyzing segregating ratios obtained from the phenotypic characterization of the F_2_ population and two identical sets of 30 F_2:3_ families. The two identical samples of 30 F_2:3_ families were tested independently with races 23 and 3481, and the degree of recombination between the putative genes was calculated by comparing the results of each pair of F_2:3_ samples tested with these two races. The cosegregatiom test used F_2:3_ samples of the families and was performed based on the facts that each F_2:3_ family genetically represents its respective F_2_ ancestor and that each F_2:3_ family can be randomly divided into two or more replicated samples.

### Inoculation and disease evaluation

The inoculum preparation; the inoculation of AC, 12 anthracnose differential cultivars, and seven additional Andean landraces with 15 races of *C*. *lindemuthianum*; and the inoculation of the resistant and susceptible parents, the F_2_ and F_2:3_ plants from crosses mentioned in the population development section above, and control cultivars with race 3481 were conducted following the methodologies described by Pastor-Corrales et al [43–45]. Several cultivars differentially resistant to race 3481, Kaboon (resistant), TO (susceptible), TU (resistant) and G 2333 (susceptible), were used as internal controls for successful inoculation. For race 23, the cultivars Widusa, Michigan Dark Red Kidney, Perry Marrow and Michelite were used as susceptible controls. In relation to the spectrum of resistance in AC, one resistant cultivar and one susceptible cultivar were used as controls for each race. The plants were evaluated for their reaction to each race of *C*. *lindemuthianum* using a severity scale from 1 to 9, where a score of 1 to 3 is considered resistant, and a score of 4 to 9 is considered a susceptible reaction [46]. Isolates from *C*. *lindemuthianum* races were obtained from the mycology collection of Nupagri. Segregant population development was performed at Nupagri. Phenotypic evaluations were conducted at Nupagri and at the Soybean Genomics and Improvement Laboratory, ARS-USDA, Beltsville Agricultural Research Center, Beltsville, Maryland. All molecular analyses were conducted in Beltsville. Seeds of the cultivars were obtained from the Nupagri Common Bean Germplasm Bank.

### Bulk segregant analysis and single nucleotide polymorphism genotyping

Newly emerged trifoliate leaves from each of the F_2_ plants from the AC × PI 207262 cross were collected. Total genomic DNA was isolated using the method described by Lamour and Finley [47] for *Phytophthora capsici*, adapted for common bean. Results from the phenotypic evaluations of F_2:3_ families derived from the AC × PI 207262 cross were used to identify homozygous resistant and homozygous susceptible F_2_ plants. Two contrasting DNA bulks were constructed by pooling equal volumes of fluorometrically standardized DNA from eight F2 plants that were homozygous RR for the resistant genotype and rr for the susceptible genotype [48]. The resistant and susceptible DNA pools were used for bulked segregant analysis [48] for the identification of markers potentially linked to the Co-AC-resistance gene. All markers were tested on the parental plants and the resistant and susceptible bulks”. The susceptible and resistant bulks and DNA from each of the AC and PI 207262 parents were screened with 5,398 SNP markers with the Illumina BeadChip BARCBEAN6K_3 following the Infinium HD Assay Ultra Protocol (Illumina, Inc. San Diego, CA) [49]. The results obtained on the BeadChip were visualized by fluorescence intensity using the Illumina BeadArray Reader, and alleles were called using Illumina GenomeStudio V2011.1 (Illumina, San Diego, CA). The allele calls were visually inspected, and errors in allele calling were corrected manually. SNPs were considered associated with the *Co-AC locus* when they were polymorphic between the AC (resistant) and PI 207262 (susceptible) parents, and the resistant and susceptible bulks clustered tightly with the resistant and susceptible parents, respectively.

### Developing simple sequence repeats linked to the *Co-AC locus*

Sequence fragments containing SNPs associated with the *Co-AC* resistance *locus* were used in the development of SSR markers. These sequences were aligned to the common bean genomic DNA sequence (*Phaseolus vulgaris* v1.0) at Phytozome, DOE, and JGI (http://www.phytozome.net) by using standalone Megablast [50] at a stringency of W = 50 and p = 95 to determine the genomic positions of the SNPs. Each sequence scaffold to which a SNP-containing sequence had aligned was then screened for the presence of SSRs using the Perl script “MISA” as described by Song et al. [51,52]. Primers were designed for the flanking sequences of the SSRs using Primer3 [53]. All the primers were designed between the genomic regions 48,448,199 bp to 50,301,592 bp (Pv01), according to Bulk Segregant Analysis (BSA) (S1 Table). The polymorphism and quality of the SSR markers were first tested using DNA from the AC (R) and PI 207262 (S) parents. The primers that were found to be polymorphic between the parents were used to analyze the F_2_ population of AC × PI 207262. PCR was performed containing 5 ng of genomic DNA, 0.25 μM of forward and reverse primers, 1X PCR Buffer (200 mM Tris-HCl (pH 8.0), 500 mM KCl, 2 mM each dNTP, 10% glycerol, 15 mM MgCl_2_, 20 ng μL^-1^ of single-stranded binding protein), and 0.1 unit of Taq DNA polymerase. The PCR thermocycling parameters were as follows: 3 min at 92°C; 38 cycles of 50 s at 92°C, 45 s at 58°C and 45 s at 72°C; followed by a 5-min extension at 72°C and hold at 10°C. The PCR products were resolved on 2–3% agarose gels (Agarose SFR, Amresco, IL, USA) prepared with TBE 1X buffer and stained with 1 μg.mL^-1^ ethidium bromide.

### Development of SNP markers linked to the *Co-AC locus*

A subset of the positively identified SNP markers from the BSA were selected for genotyping the F_2_ populations from the AC × PI 207262 and AC × G 2333 crosses. Genotyping was accomplished using kompetitive allele specific PCR (KASP) assays. The KASP primer sequences were designed using Primer3 [52,53], and the KASP reactions were conducted following the manufacturer's instructions in 10 μL reactions with 5 μL of 2X premade KASP master mix (LGC, Middlesex, UK), 0.14 μL of primer mix (Sigma-Aldrich, St. Louis, USA), and 20–40 ng of genomic DNA. PCR parameters were performed as described by LGC on standard thermocycling machines using white semiskirted polypropylene 0.2 mL 96-well PCR plates (USA Scientific) and sealed with Microseal B (Bio-Rad, Hercules, CA, USA). After PCR amplification was completed, the PCR plates were scanned using the Mx3000P qPCR machine (Agilent, Santa Clara, CA), and allele calls for each genotype were obtained using KlusterCaller software (LGC, Middlesex, UK).

### Construction of genetic linkage map around the *Co-AC locus*

The genetic distances between the SSRs, KASPs, and the *Co-AC locus* in the two F_2_ populations from the AC × PI207262 and AC × G 2333 crosses were estimated using the software JoinMap 4.0 [48]. The default settings of a regression mapping algorithm based on the Kosambi map function were chosen to define linkage order and distances in centimorgans (cM). A minimum logarithm of odds (LOD) ≥3.0 and a maximum distance of ≤50 cM were used to test linkage among the markers.

### Haplotype analysis around the *Co-AC locus*

Haplotype analysis was performed in the genomic region flanked by SNP markers SS56 (49,895,862 bp) and SS92 (50,527,176 bp). Eighteen diverse bean cultivars including the Mesoamerican C20, Matterhorn, Stampede, T39, Michelite, UC White, Sierra, UI 906, Laker, Buckskin, Cornell 49242, and BAT 93, and the Andean Jalo EEP 558, Fiero, Lark, California Early Light Red Kidney (CELRK), Kardinal, Red Hawk, re-sequenced by Song et al. [49], were used for haplotype analysis. Andean and Mesoamerican haplotypes were built, and SNPs that differentiated the common bean gene pools were chosen for KASP marker designing. SNP tables were handled using Microsoft Excel and haplotypes were identified by visual inspection across the flanking region, choosing haplotypes Andean/Mesoamerican. Twenty SNPs were chosen between the physical genomic region of 50,398,715 and 50,667,035 bp on Pv01 and converted into KASP primers. When KASP markers were polymorphic between the AC and PI 207262 parents, KASP markers were used to genotype F_3_ recombinants plants.

### Target resequencing for SNP marker development

To find additional SNPs in the genomic region between the KASP markers SS102 (50,377,247 bp) and SS95 (50,442,472 bp), eight intergenic genomic regions of approximately 1 Kbp in size each were selected for Sanger sequencing. The eight targeted regions were selected using the reference genome of common bean, assembly v1.0 [35]. Primers were designed using the software Primer-BLAST [51]. DNA from the parents AC (R) and PI 207262 (S) was used to obtain PCR amplicons. PCR was carried out using 5 ng of genomic DNA, 0.25 μM of forward and reverse primers, 1X PCR Buffer, and 1 unit of Taq DNA polymerase. The PCR thermocycling parameters were as follows: 3 min at 94°C; 38 cycles of 50 s at 94°C, 45 s at 57°C—61°C and 45 s at 72°C; followed by a 5-min extension at 72°C and hold at 4°C. The amplicons were separated using 1.2% agarose gel electrophoresis and visualized under ultraviolet light. The amplicons corresponding to each targeted region from each parent were purified using the QIAquick^®^ kit (Qiagen, Germantown, MD) following the manufacturer’s instructions. Purified samples were quantified using the Qubit and sent for Sanger sequencing to the Centro de Estudos do Genoma Humano e Células Tronco (CEHG-CEL) of the Universidade de São Paulo (USP), São Paulo State, Brazil. The sequenced reads were analyzed using v5.4.6 (Gene Codes Corporation, Ann Arbor, MI, USA). Then, the sequences from each sample were blast-compared using the software NCBI-BLAST to verify if the sequenced amplicon corresponded to the targeted region. The SNPs that were polymorphic between AC and PI 207262 in each targeted region were identified by alignment comparison and converted into KASP markers as previously described. Successfully amplified KASP markers were used to genotype the F_3_ recombinant plants.

### Fine mapping of the *Co-AC locus* using F_3_ plants

A total of 57 F_2:3_ families derived from the AC × PI 207262 cross were selected for fine mapping of the *Co-AC locus*. These 57 F_2:3_ families were heterozygous or recombinant around the *Co-AC locus* based on the alleles detected between markers SS56 and SS92 in the F_2_ plants. Three additional homozygous susceptible families were included in the phenotyping process as internal controls. The number of plants per family varied from 1 to 15 depending on the viability of their seeds. A total of 660 F_3_ plants were phenotyped for their reactions to race 3481 of *C*. *lindemuthianum* as described above in the Methods section. DNA isolation was also performed as described above. The 660 F_3_ plants were genotyped with the SNP markers SS56 and SS92, which flank the *Co-AC locus*. F_3_ plants showing recombination between markers SS56 and SS92 were selected for additional genotyping with newly designed SNP markers to narrow the region of the *Co-AC locus*. The reference genome sequence G19833 common bean version 1.0 available in Phytozome.org was used for *in silico* browsing of the candidate genes and annotations [35].

### Results

The resistance spectra for AC, 12 anthracnose differential cultivars and seven Andean cultivars to are presented in Table 1. None of the 20 cultivars evaluated were resistant to all 15 races. AC was resistant to 13 races, seven Mesoamerican (9, 65, 73, 89, 1545, 2047, and 3481) and six Andean (2, 7, 19, 23, 39, and 55), but AC was susceptible to two Mesoamerican races (449 and 453). The Mesoamerican cultivar G 2333, which has three anthracnose-resistance genes (*Co-3*^*5*^, *Co-4*^*2*^, *Co-5*^*2*^), was susceptible to the Mesoamerican race 3481. G 2333 has been considered resistant to several hundred races of the anthracnose pathogen worldwide [3,12,13,54,55]. Except for G 2333, the other differential cultivars were susceptible to race 2047. On the other hand, AC and five Andean landraces were resistant to race 2047. Thus, the results of this study underscored the comprehensive resistance of AC to a broad diversity of Mesoamerican and Andean races of *C*. *lindemuthianum*.

**Table 1:** Reactions of 12 differential cultivars and of eight Andean common bean cultivars to Mesoamerican and Andean races of *Colletotrichum lindemuthianum*.

		^2^Gene	Genes		^1^Races of *Colletotrichum lindemuthianum*													
		pool		2	7	9	19	23	39	55	65	73	89	449	453	1545	2047	3481
**Differential cultivars**																		
	Michelite	M	*Co-11*	R	S	S	S	S	S	S	S	S	S	S	S	S	S	S
	^3^MDRK	A	*Co-1*	S	S	R	S	S	S	S	R	R	R	R	R	R	S	R
	Perry Marrow	A	*Co-1*^*3*^	R	S	R	R	S	S	S	R	R	R	R	S	R	S	R
	Cornell 49–242	M	*Co-2*	R	R	S	R	R	R	R	R	S	S	R	R	S	S	S
	Widusa	A	*Co-1*^*5*^	R	R	R	S	S	R	S	R	R	S	R	R	R	S	S
	Kaboon	A	*Co-1*^*2*^	R	R	R	R	R	S	S	R	R	R	R	R	R	S	R
	Mexico 222	M	*Co-3*	R	R	R	R	R	R	R	S	S	S	S	S	R	S	R
	PI 207262	M	*Co-4*^*3*^*/Co-3*^*3*^	R	R	R	R	R	R	R	R	R	R	S	S	R	S	S
	TO	M	*Co-4*	R	R	R	R	R	R	R	R	R	R	S	S	R	S	S
	TU	M	*Co-5*	R	R	R	R	R	R	R	R	R	R	R	R	S	S	R
	AB 136	M	*Co-6*	R	R	R	R	R	R	R	R	R	R	R	R	S	S	S
	G 2333	M	*Co-4*^*2*^*/Co-5*^*2*^*/Co-3*^*5*^	R	R	R	R	R	R	R	R	R	R	R	R	R	R	S
**Andean Sources of Resistance**^3^																		
	Amendoim Cavalo	A	*Co-AC*	R	R	R	R	R	R	R	R	R	R	S	S	R	R	R
	AND 277	A	*Co-1*^*4*^	R	S	R	S	R	S	R	R	R	R	R	R	R	R	R
	Jalo Vermelho	A	*Co-12*	R	S	R	S	R	S	R	R	S	R	R	R	R	S	S
	Jalo Listras Pretas	A	*Co-13*	S	S	R	S	S	S	S	R	R	R	S	S	R	S	R
	Pitanga	A	*Co-14*	R	S	R	R	R	S	R	R	R	S	S	S	S	R	S
	Corinthiano	A	*Co-15*	R	S	S	S	R	NE	S	R	R	R	R	S	R	R	S
	Paloma	A	*Co-Pa*	S	S	S	S	R	R	R	R	R	S	S	S	R	R	R
	Jalo Pintado 2	A	*Co-JP*	R	R	R	S	S	S	S	R	R	S	R	R	R	R	R

^1^Nine Mesoamerican (9, 65, 73, 89, 445, 453, 1545, 2047 and 3481) and six Andean (2, 7, 19, 23, 39, and 55) races of *Colletotrichum lindemuthianum*.

^2^A = Andean and M = Mesoamerican; ^3^MDRK = Michigan Dark Red Kidney.

^3^Adapted Castro et al. (2017).

### Inheritance of the resistance *locus Co-AC*

The inheritance of resistance in AC was determined using the reactions of 302 F_2_ plants from two populations inoculated with race 3481 of *C*. *lindemuthianum*; 113 F_2_ plants were from the AC × PI 207262 cross, and 189 F_2_ plants were from the AC × G 2333 cross. AC was resistant, while PI 207272 and G 2333 were susceptible to race 3481. A total of 83 F_2_ plants from the AC × PI 207262 cross were resistant, and 30 plants were susceptible, consistent with a 3 resistant: 1 susceptible segregation ratio (*χ*^*2*^ = 0.145, *P value* = 0.704) (Table 2). From the AC × G 2333 cross, 144 F_2_ plants were resistant, and 45 plants were susceptible, which also fitted a 3 resistant: 1 susceptible ratio (*χ*^*2*^ = 0.143, *P value* = 0.705) (Table 2). Overall, the inheritance of resistance results was consistent with a 3 resistant: 1 susceptible ratio in these two populations, indicating monogenic dominant inheritance of resistance via the *Co-AC locus* in AC.

**Table 2:** Inheritance of anthracnose resistance in the Andean common bean cultivar Amendoim Cavalo (AC) using two mapping populations; AC × PI 207262 and AC × G 2333.

		Observed Ratio (3:1)		Expected Ratio (3:1)		Total	χ^*2*^	*P-value*
	Generation	R	S	R	S
AC	RP^1^	34	0	-	-	34	-	-
PI 207262	SP^2^	0	14	-	-	14	-	-
G 2333	SP^2^	0	18			18		
AC × PI 207262	F_2_	83	30	84.75	28.25	113	0.145	0.704
AC × G 2333	F_2_	144	45	141.75	47.25	189	0.143	0.705

^1^Resistant parent

^2^Susceptible parent.

R = Number of resistant plants; S = Number of susceptible plants.

### Cosegregation analysis using *Colletotrichum lindemuthianum* Races 23 and 3481

A total of 562 F_3_ plants from 30 F_2:3_ families derived from the AC × Widusa cross were separated into two groups that were inoculated separately, one with race 23 and the other with race 3481 of *C*. *lindemuthianum*. These two groups exhibited identical cosegregation of resistance/susceptibility to both races. All plants that were resistant to race 23 were also resistant to race 3481, whereas all plants that were susceptible to race 23 were also susceptible to race 3481. No recombinants were observed in these F_2:3_ families. The observed cosegregation ratio of 8RR:15Rr:7rr individuals (*χ*^*2*^ = 0.067, *P value* = 0.967) fitted the expected 1RR:2Rr:1rr ratio.

### BSA and SNP genotyping using the BARCBean6K_3 BeadChip

Based on the BSA, 27 SNPs were associated with the anthracnose-resistance *locus* of the Andean common bean landrace AC, provisionally named *Co-AC* (S1 Table). These SNPs distinguished the susceptible PI 207262 parent and the susceptible bulk from the resistant AC and the resistant bulk. According to the genetic linkage map created by Song et al. [48], these 27 SNPs were distributed in a genomic region from 58.09 to 67.61 cM, located on the lower end of the common bean chromosome Pv01 (S1 Table). The physical locations of the 27 SNPs associated with the *Co-AC locus* were between ss715646585 (48,448,199 bp) and ss715645251 (50,301,592 bp) in a genomic region spanning a total of 1.85 Mbp.

### Mapping of the *Co-AC locus*

**Fig 1:**
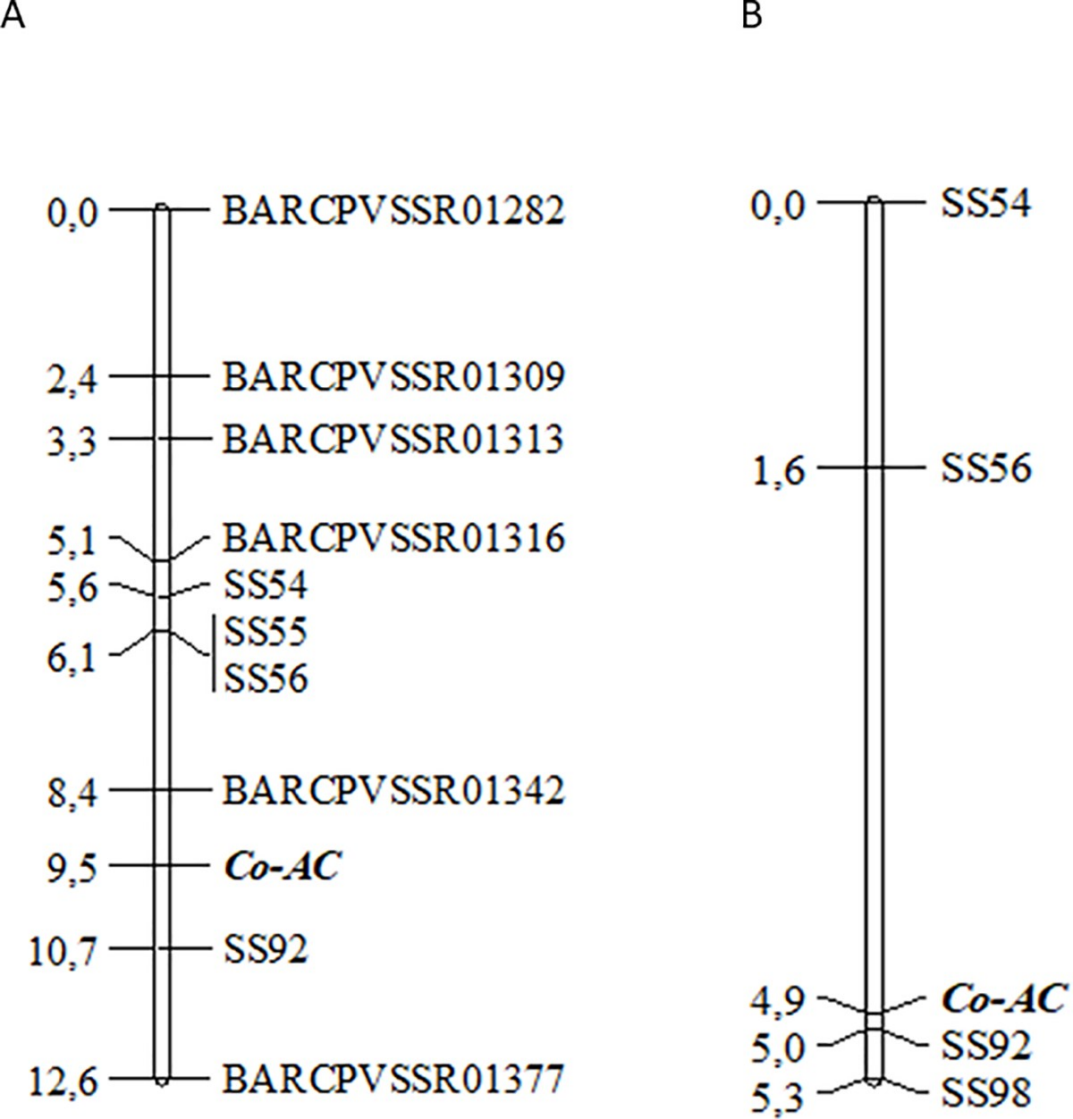
Genetic linkage maps of the anthracnose résistance *locus Co-AC locus* on chromosome Pv01 of *Phaseolus vulgaris* obtained using two different F_2_ mapping populations. Fig 1A: acquired using 109 F_2_ plants from Amendoim Cavalo × PI 207262 cross. Fig 1B: obtained using 187 F_2_ plants from Amendoim Cavalo × G2333 cross. Linkage maps were constructed using simple sequence repeats (SSRs) and SNP KASP markers. The *Co-AC locus* was flanked by SNP KASP markers ss56 and ss92 in both F_2_ mapping populations.

The large genomic region containing the 27 SNPs associated with the anthracnose-resistance *locus Co-AC* was targeted for SSR and SNP development. A total of 28 SSR markers were developed. These markers were located between 48,448,199 and 50,301,592 bp (S1 Table) on chromosome Pv01. Six of the 28 SSRs were polymorphic between the resistant AC and the susceptible PI 207262 parents (S2 Table). These markers, which showed unequivocal allele separation in an agarose gel, were used to map the *Co-AC locus* in the F_2_ population from the AC × PI 207262 cross. In addition, five positively associated SNPs from the BSA were selected and converted into KASP markers (S3 Table). All five KASP markers (SS54, SS55, SS56, SS92, and SS98) showed clear separation of the three clusters (2 homozygous and 1 heterozygous) and were polymorphic between the AC and PI 207262 parents. Four of the five KASP markers (SS54, SS55, SS56, and SS92) were used to more precisely map the position of the *Co-AC locus*. Linkage analysis in the F_2_ population from the AC × PI 207262 based on genotyping with six SSRs and four KASP markers showed that the *Co-AC locus* was flanked by the SSR marker BARCPVSSR01342 (50,038,283 bp) and KASP marker SS92 (50,527,176 bp) on chromosome Pv01 (Fig 1A). The distance from the *Co-AC locus* to the flanking SSR and KASP markers was 1.1 cM and 1.2 cM, respectively (Fig 1A). Additionally, 189 F_2_ plants from the AC × G 2333 cross were used to map and confirm the genetic position of the *Co-AC locus* on the map generated using the AC × PI 207262 cross. Four KASP markers (SS54, SS56, SS92, and SS98) were used to genotype the F_2_ population from the AC x G 2333 cross. The *Co-AC locus* was positioned between the KASP markers SS56 (49,895,862 bp) and SS92 (50,527,176 bp) at a distance of 3.3 cM from SS56 and 0.1 cM from SS92 (Fig 1B). The linkage analysis based on the AC × G 2333 cross confirmed the position of the *Co-AC locus* observed in the first AC × PI 207262 cross; that is, the *Co-AC locus* was flanked by the KASP markers SS56 and SS92 (Fig 1A).

### Fine mapping of the *Co-AC* resistance *locus*

**Fig 2:**
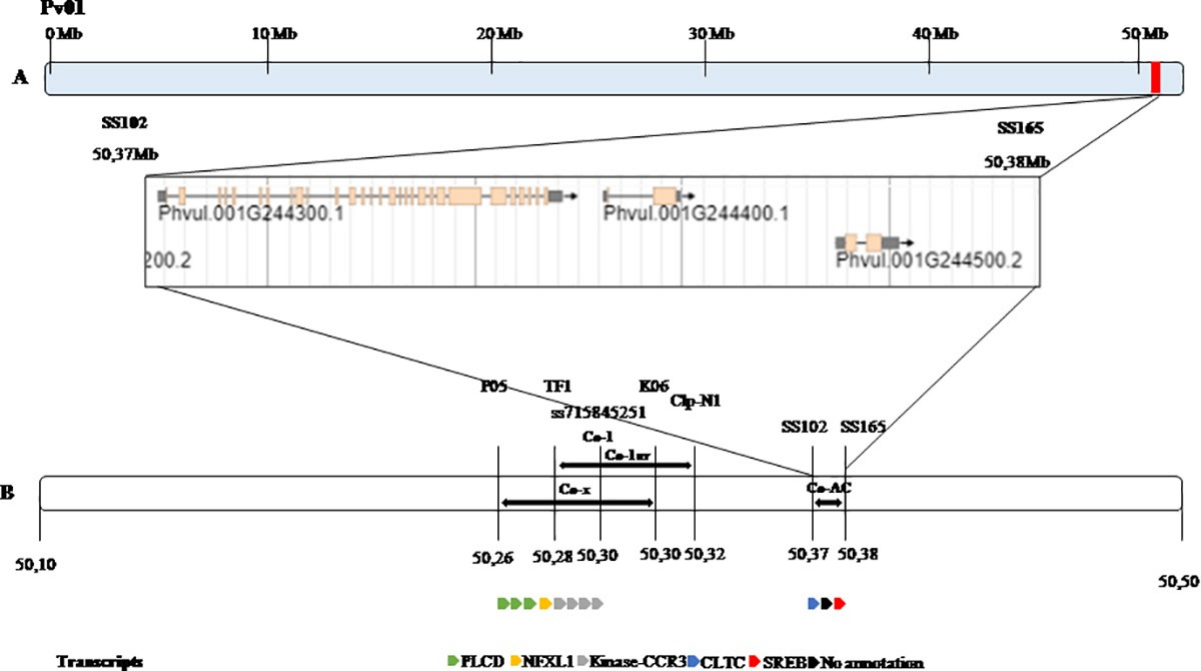
Fine mapped region for Amendoim Cavalo resistance *locus Co-AC*. Fig 2A. Chromosome Pv01 with *Co-AC* region highlighted in red at the end of the chromosome. Three predicted genes are within *Co-AC* region flanked by the markers SS102 and SS165. Fig 2B. Comparison between the physical position (Mb) of the *Co-AC locus*, *Co-x* [33], *Co-1*, and *Co-1*^*HY*^ [8] and predicted transcripts for each gene region. Color codes represent predicted gene functions. Orange represent phosphatidylinositol phospholipase C, delta (PLCD) protein, purple represent NF-X1 type zinc finger protein (NFXL1), green represent serine/threonine-protein kinase- like protein CCR3-related protein (Kinase-CCR3), blue represent clathrin heavy chain protein (CLTC), red represent sterol regulatory element binding protein (SREB) and black represent protein with unknown function. The genomic region between these markers is indicated by the lower bar and cover around 9,45 Kbp of the genome.

The flanking KASP markers SS56 and SS92 were mapped at 3.4 cM and 1.2 cM from the *Co-AC locus*, respectively, in the AC × PI 207262 cross (Fig 1A and Fig 2A). These two flanking KASP markers were chosen to genotype each individual of the 57 F_2:3_ families (with 660 F_3_ plants) from the AC × PI 207262 cross. The SNP markers SS56 and SS92 spanned a physical genomic region of 631 Kbp of chromosome Pv01 according to the reference genome G 19833 (Fig 2A). A total of 77 F_3_ plants out of the 660 F_3_ plants screened with the markers SS56 and SS92 contained recombinant events across the *Co-AC locus*. These 77 F_3_ plants were selected for subsequent genotyping with nine other SNP markers. Among the 77 recombinant F_3_ plants, 54 plants were informative, and sixteen informative recombination events were observed (S4 Table). Based on these recombination events, the *Co-AC locus* was fine-mapped between the SNP markers SS102 and SS165 (S4 Table and Fig 2A). The physical region between these two SNP markers flanking the *Co-AC locus* was 9.5 Kbp in size.

### Candidate genes for *Co-AC*

The new flanking markers for the *Co-AC locus* were located between positions 50,377,247 bp (SS102) and 50,386,692 bp (SS165), at the end of chromosome Pv01, based on the *in silico* analysis of the reference genome G19833 version 1.0 [35]. The genomic region between these two markers flanking the anthracnose-resistance *locus* in *AC* is approximately 9.45 Kbp in size (Fig 2). This region contains three predicted genes based on the common bean reference genome version 1.0 (Table 3). These three hypothetical proteins were Phvul.001G244300 (at position 50,365,442–50,377,656 bp), Phvul 001G244400 (50,378,875–50,381,218 bp), and Phvul. 001G244500 (50,385,367–50,387,821 bp). Two of the three hypothetical proteins contained known domains, which were described as a Clathrin Heavy Chain (CLTC) for Phvul.001G244300 and a helix-loop-helix for Phvul.001G244500 (Table 3). No predicted function was identified for Phvul.001G244400. These genes are likely candidates for the *Co-AC* anthracnose-resistance *locus*. The putative homologous of these genes in the plant model *A*. *thaliana* were identified for further functional reference (Table 3).

**Table 3:** Gene models found in delimitated region Amendoim Cavalo resistance *locus* against anthracnose was fine-mapped, onthology to *A*. *thaliana* and annotation.

Gene Model	Homolog in *A*. *thaliana*	E-value*	Identity*	Functional annotation on TAIR**	Functional annotation on Phytozome***
in *P*. *vulgaris*					
Phvul.001G244300	AT3G11130	0	92.40%	Clathrin heavy chain 1	Clathrin Heavy Chain (CLTC) involved in plant defense signaling
Phvul.001G244400	AT3G56010	8 x 10^−44^	54.17%	Transmembrane protein	Unknown function
Phvul.001G244500	AT2G40200	3 x 10^−47^	41.83%	Basic helix-loop-helix (bHLH) DNA-binding superfamily protein	Helix-loop-helix DNA-binding domain with possible transcription function

*E-values and Identity for BLASTp analysis performed on NCBI (National Center for Biotechnology Information; https://www.ncbi.nlm.nih.gov).

** Functional gene annotation resource: TAIR—The Arabidopsis Information Resource (https://www.arabidopsis.org).

*** Functional gene annotation resource: Phytozome—Common bean reference genome v1.0 (https://phytozome.jgi.doe.gov#).

Interesting, the marker SS102 (50,377,247) is physically mapped at 409 bases upstream the stop codon of the Phvul.001G244300, at the last predicted intron (Fig 2). As displayed in the Fig 2, the marker SS165 is located at 601 bases from the stop codon of the predicted gene Phvul.001G244500, providing a mutation in the last exon of this gene, that encodes a putative Helix-loop-helix DNA-binding domain.

### Discussion

An important objective of this study was to confirm the resistance of the Andean common bean AC to the high virulence diversity of *C*. *lindemuthianum*. To that end, the reactions of AC to 15 races of this pathogen, 9 Mesoamerican and six Andean, were compared to the reactions of 12 anthracnose differential cultivars and to a group of seven Andean landraces known to possess reported anthracnose-resistance genes [18–20,26,27,29]. A total of 85% of the Mesoamerican and Andean cultivars used in this study were susceptible to three or more races (up to 14 of the 15 races) of *C*. *lindemuthianum*. AC was resistant to 13 races, seven Mesoamerican (9, 65, 73, 89, 1545, 2047, and 3481) and six Andean (2, 7, 19, 23, 39, and 55) races. The highly resistant Mesoamerican differential cultivar G 2333, known to carry the anthracnose-resistance genes *Co-4*^*2*^, *Co-5*^*2*^, *Co-3*^*5*^ [27,54], was susceptible to one race, the Mesoamerican race 3481, to which AC was resistant. G 2333 has been evaluated as resistant to several hundred races of the anthracnose pathogen [3,12,13,54,55]. Thus, these results underscore the comprehensive broad-spectrum resistance of AC to Mesoamerican and Andean races of *C*. *lindemuthianum*. The resistance of AC was broader than the resistance of the four Andean and six Mesoamerican differential cultivars. It is also significant that AC was resistant to Mesoamerican races 1545, 2047, and 3481. AC was resistant to Mesoamerican races that have been reported in Brazil, Argentina, Colombia, all the countries of Central America, Mexico, the Caribbean, USA, Canada, Africa and Europe. Similarly, AC was resistant to Andean races that have been reported to occur in Argentina, Brazil, Peru, Ecuador, Colombia, Mexico, the Caribbean, USA, Canada, and South Africa [1,10–14,44]. The resistance of AC to the Andean races 2, 7, 19, 23, and 55 and to the Mesoamerican races 9, 65, 73, and 89 is of importance to common bean breeding programs in Brazil, where these races have been reported frequently [12,15,18]. AC was susceptible to the Mesoamerican races 449 and 453 from Mexico, which were almost identical in their virulence spectra on the 12 differential cultivars. However, several Mesoamerican and Andean cultivars were resistant to races 449 and 453. The unique broad-spectrum anthracnose resistance of AC, seldom observed in other Andean common bean cultivars, could be very useful to protect Mesoamerican common bean cultivars that harbor broad-spectrum anthracnose resistance but remain susceptible to highly virulent races of Mesoamerican origin. Remarkably, the resistance of AC can also protect Andean bean cultivars from highly virulent Andean races of *C*. *lindemuthianum*, such as races 7, 39, and 55.

Segregating F_2_ populations from the cross between the resistant AC and the susceptible differential cultivars PI 207262 and G 2333, when inoculated with race 3481, revealed an anthracnose-resistance *locus* in AC, provisionally called *Co-AC*, which segregated as a monogenic dominant *locus*. Nanami and collaborators previously reported that anthracnose resistance in AC was conditioned by a single dominant gene [42]. These authors performed several allelism tests including crosses between AC and the cultivars Michigan Dark Red Kidney (*Co-1*), Jalo Vermelho (*Co-12*), Jalo Listras Pretas (*Co-13*), Pitanga (*Co-14*), and Paloma (*Co-Pa*), which have anthracnose-resistance *loci* located on Pv01, and concluded that based on these allelism tests, the anthracnose resistance of AC was independent of the anthracnose-resistance genes found in the other five cultivars used in the tests.

In the present study, we conducted a segregation analysis of two samples from 30 F_2:3_ families from the AC (R) × Widusa (S) cross. One sample was inoculated with race 23 and the other with race 3481 of *C*. *lindemuthianum*. In progeny tests, the two F_2:3_ samples showed the same resistance and susceptibility reactions. Plants that were resistant or susceptible to race 23 were also resistant or susceptible to race 3481. No recombination was observed between the two putative genes conferring resistance to races 23 and 3481. These results indicate that a single dominant gene controls resistance to races 23 and 3481 in AC.

Utilizing BSA and SNP genotyping of F_2_ and F_2:3_ families, we positioned the *Co-AC locus* in a significantly smaller genomic region of 9,445 bp flanked by the SNP markers SS102 (50,377,247 bp) and SS165 (50,386,692 bp). When our results were compared to those of others by integrating linkage maps (Fig 2), we found different markers, such as SS102 and SS165. This is the smallest reported genomic region containing an anthracnose-resistance *locus* (*Co-AC*); it is considerably smaller than the genomic regions previously reported for the fine mapping of the *Co-x* and *Co-1*^*HY*^ *loci* [8,34]. The *Co-x locus* was fine-mapped to a 58 Kbp region flanked by the markers P05 (50,264,307 bp) and K06 (50,322,583 bp) [34]. Likewise, the *Co-1*^*HY*^ *locus* was fine-mapped to a 46.6 Kbp region between the markers TF1 (50,286,325 bp) and Clp-N1 (50,332,945 bp) [8]. The fine-mapped positions of the *Co-x* and *Co-1*^*HY*^ *loci* overlapped, but the position of *Co-AC* was fine-mapped approximately 54.6 Kbp and 44.3 Kbp downstream of *Co-x* and *Co-1*^*HY*^, respectively [8,34]. Based on the physical positions of the abovementioned markers, *Co-AC* is clearly located at a distance of 127,161 bp from *Co-1*^*4*^.

Furthermore, Zuiderveen et al. [30] performed a genome-wide association study (GWAS) using the Andean Diversity Panel (ADP) and identified that the *Co-1* allele is closely linked to SNPs ss715645258 and ss715645251 on Pv01. *Co-AC* was also positioned 75.8 Kbp downstream of the *Co-1* allele, which was mapped to a 145.6 Kbp genomic region (50,155,927–50,301,532 bp) obtained using association mapping and validated with the recombinant inbred line (RIL) population from the Jaguar x Puebla 152 cross [32] (Fig 2B). These results suggest that the *Co-AC locus* is different from the *Co-1*, *Co-1*^*4*^, Co*-1*^*HY*^ and *Co-x loci*.

Fine mapping of the *Co-AC locus* resulted in the identification of three candidate genes, the smallest number of candidate genes yet reported for any anthracnose disease resistance gene in common bean. Using *in silico* analysis based on the G19833 Andean reference genome version 1.0, no classical NB-LRR or kinase disease resistance gene models were identified in the fine-mapped region containing the *Co-AC locus* (Table 3). However, the candidate gene Phvul.001G244300 might play a role in plant defense signaling. This candidate gene is annotated as a hypothetical protein with a Clathrin heavy chain domain (Table 3). Recently, it has been proposed that an important step in the plant immunity response against plant pathogens is mediated by endocytosis of the activated plant cell surface receptor after pathogen recognition [56]. Clathrin-mediated endocytosis (CME) is the major route of endocytosis known in plants [57], and Phvul.001G244300 might be involved in the defense response of the AC landrace when infected by *C*. *lindemuthianum*. Functional characterization of these cellular events during pathogen invasion is needed.

Among the candidate genes identified in the *Co-AC* resistance *locus*, the Phvul.001G244300 and Phvul.001G244500 genomic sequences contain the flanking markers for this *locus* (SS102 and SS165, respectively; Fig 2 and Table 3). Phvul.001G244300 is a homolog of *A*. *thaliana* Clathrin heavy chain 1 with 92.40% identity and an e-value of zero; this protein supports specific functions in multiple cell types and has shown that clathrin is important for stomatal regulation [58]. In 1976, Pearse isolated clathrin for the first time and reported it as a protein that plays a major role in the formation of coated vesicles [59]. Endocytosis in plants has an essential role not only in basic cellular functions but also in growth and development, hormonal signaling and communication with the environment, including nutrient delivery, toxin avoidance, and pathogen defense [60]. Indeed, the major endocytic mechanism in plants depends on the coat protein clathrin [61]. Expression of clathrin heavy chain gene, ZmCHC1, in maize was observed significantly up-regulated by salicylic acid (SA) or abscisic acid (ABA) suggesting that ZmCHC1 is involved in SA signaling pathway in defense responses [60]. In the present study, although the Phvul.001G244400 gene did not encode a protein, it is important to note that this gene is homologous to the AT3G56010 gene of *A*. *thaliana* with 54.17% identity and an e-value = 8 × 10^−44^, and the *A*. *thaliana* gene encodes a transmembrane protein.

Previous studies have provided evidence for the existence of at least one interacting signaling molecule in Arabidopsis: PIF3, in which photoactivated phytochrome has been shown to bind specifically to DNA-bound PIF3 [62]. Toledo-Ortiz and collaborators [63] reported that ABS5 encodes a protein of 368 amino acids, and protein sequence analysis revealed that ABS5 is likely a putative transcription factor belonging to the basic helix-loop-helix (bHLH) family. The authors noted that in Arabidopsis, at least 147 members have been identified in the bHLH family.

Notably, Phvul.001G244500 is a homolog of a Basic helix-loop-helix (bHLH) DNA-binding superfamily protein in Arabidopsis with 41.83% identity and an e-value of 3 × 10^−47^. Therefore, Phvul.001G244500 could affect the regulatory interactions between stem cells and organizing cells in the root and shoot apical meristems [64]. A basic helix-loop-helix (BHLH) transcription factor homolog of brassinosteroid enhanced expression interacting with IBH1 (HBI1) was identified as a negative regulator of pathogen-associated molecular pattern (PAMP)-triggered immunity (PTI) signaling in *Arabidopsis thaliana* [65]. IBH1 overexpression led to reduced PAMP-trigged responses and increased susceptibility to the bacterium *Pseudomonas syringae*. [65].

Thus, we also highlight here the gene model Phvul.001G244300, encoding Clathrin heavy chain 1, which supports specific functions in stomatal regulation and might play a role in plant defense signaling. Because the *Co-AC* resistance *locus* is linked in *cis*, it can be selected with great efficiency using molecular markers. These results will be very useful for breeding programs aimed at developing bean cultivars with anthracnose resistance using marker-assisted selection.

## Conclusions

Our results revealed the broad-spectrum resistance of the Andean cultivar AC to 15 highly diverse Mesoamerican and Andean races of C. *lindemuthianum*. The unique resistance of AC could be highly useful for protecting Mesoamerican common bean cultivars with broad-spectrum anthracnose resistance. AC was susceptible to two Mesoamerican races (449 and 453) from Mexico that were very similar in virulence. Several cultivars of Mesoamerican and Andean origin are resistant to these two races. The resistance of AC can also protect Andean bean cultivars from virulent Andean races. Fine mapping positioned the *Co-AC locus* in a small genomic region of 9.4 Kbp via the identification of two tightly linked markers. Haplotype examination of the SS102 and SS165 markers detected two major haplotypes that corresponded to the Mesoamerican and Andean gene pools of common bean. Fine mapping of the *Co-AC locus* resulted in the identification of three candidate genes, none of which were classical NB-LRR or kinase genes. However, the candidate gene Phvul.001G244300 might play a role in plant defense signaling.

## Supporting information

S1 Table Single Nucleotide Polymorphism markers associated with the anthracnose resistance locus in Andean common bean landrace Amendoim Cavalo discovered by bulk segregant analysis and located on the lower end of chromosome Pv01 of common bean. *Genetic position based on the map developed by Song et al. [49].
(DOC)

S2 Table Simple sequence repeats marker ID, motif, size, forward primer position, and forward primer sequences on version 1.0 of the reference genome of Phaseolus vulgaris and primer sequences. (DOC)

S3 Table KASP markers used to map the Co-AC resistance locus on the Andean common bean landrace Amendoim Cavalo. (DOC)

S4 Table Genotype and phenotype of 77 F_3_ recombinant plants used for fine mapping of the Co-AC anthracnose resistance locus. The phenotype was obtained from the reaction of the 77 F_3_ plants to race 3481 of *Colletotrichum lindemuthianum*. Genotyping was achieved using the flanking KASP markers SS56 and SS92 (highlighted in orange) and the nine other KASP markers (highlighted in yellow) that enabled the positioning of the *Co-AC* locus in a 9,445 bp genomic region flanked by markers SS102 and SS165. A total of 54 out 77 F_3_ recombinant plants were assigned a recombination type from 1 to 16. AA = homozygous susceptible as PI 207262, BB = homozygous resistant, as the Amendoim Cavalo locus, AB = heterozygous resistant.
(DOC)

## Abbreviations

ADP: Andean Diversity Panel
ARS: Agricultural Research Service
BSA: bulk segregant analysis
cM: centimorgans
CNL: N-terminal coiled-coil domain
GWAS: genome-wide association study
KASP: kompetitive allele specific PCR
NB-LRR: nucleotide-binding, leucine-rich repeat
Nupagri: Núcleo de Pesquisa Aplicada à Agricultura
PCR: polymerase chain reaction
PI-PLC: phosphoinositide-specific phospholipase-C
PTK: protein tyrosine kinase
RB: resistant bulk
RIL: recombinant inbred line
SB: susceptible bulk
SNP: single nucleotide polymorphism
STK: serine threonine protein kinase
TBE: Tris-borate-EDTA
TNL: N-terminal *Toll*-interleukin-1 receptor (TIR)-like domain
UEM: Universidade Estadual de Maringá
USDA: United States Department of Agriculture
