# Supplementary material for: Fine mapping of an anthracnose-resistance locus in Andean common bean cultivar Amendoim Cavalo
Source: PLoS One. 2020 Oct 7;15(10):e0239763. doi: 10.1371/journal.pone.0239763 (PMC7540868; doi:10.1371/journal.pone.0239763)
Supplement: S1 Table — *Genetic position based on the map developed by Song et al. [49]. (DOC) [file pone.0239763.s001.doc]

**Table S1** Single Nucleotide Polymorphism markers associated with the anthracnose resistance locus in Andean common bean landrace Amendoim Cavalo discovered by bulk segregant analysis and located on the lower end of chromosome Pv01 of common bean. *Genetic position based on the map developed by Song et al. [49]

| NCBI ID | BARCBEAN6K_3 SNP id | Genetic position (cM)* | SNP physical |
| --- | --- | --- | --- |
| Code |  |  | Position |
| ss715646585 | sc00076ln674865_383201_A_G_75705477 | 58.09 | 48,448,199 |
| ss715645886 | sc00022ln1003704_463708_T_C_32965580 | - | 49,303,661 |
| ss715645859 | sc00022ln1003704_178718_A_G_32680590 | 63.93 | 49,588,715 |
| ss715645856 | sc00022ln1003704_150441_G_A_32652313 | 63.93 | 49,617,274 |
| ss715645853 | sc00022ln1003704_129339_C_A_32631211 | 63.93 | 49,637,944 |
| ss715645852 | sc00022ln1003704_109623_C_T_32611495 | 63.93 | 49,657,760 |
| ss715645935 | sc00022ln1003704_96352_G_A_32598224 | 64.12 | 49,671,031 |
| ss715645891 | sc00022ln1003704_49856_T_G_32551728 | 64.64 | 49,718,129 |
| ss715645862 | sc00022ln1003704_22533_G_A_32524405 | 64.64 | 49,742,126 |
| ss715645855 | sc00022ln1003704_14515_T_C_32516387 | 64.65 | 49,749,711 |
| ss715645288 | sc00003ln2130026_2113635_G_A_6438335 | 64.86 | 49,783,658 |
| ss715645287 | sc00003ln2130026_2105132_A_G_6429832 | 65.16 | 49,793,139 |
| ss715645286 | sc00003ln2130026_2057851_G_A_6382551 | - | 49,841,858 |
| ss715645284 | sc00003ln2130026_2037421_C_A_6362121 | - | 49,862,290 |
| ss715645280 | sc00003ln2130026_2002999_C_T_6327699 | 65.99 | 49,895,862 |
| ss715645274 | sc00003ln2130026_1923031_T_G_6247731 | 66.59 | 49,969,810 |
| ss715645269 | sc00003ln2130026_1850170_T_C_6174870 | 66.79 | 50,042,771 |
| ss715645266 | sc00003ln2130026_1827265_G_A_6151965 | 66.79 | 50,065,488 |
| ss715645263 | sc00003ln2130026_1796976_T_C_6121676 | 66.79 | 50,093,966 |
| ss715645262 | sc00003ln2130026_1791042_A_G_6115742 | 66.79 | 50,099,818 |
| ss715645260 | sc00003ln2130026_1773813_T_C_6098513 | 66.79 | 50,115,685 |
| ss715645258 | sc00003ln2130026_1733138_T_C_6057838 | 66.99 | 50,155,987 |
| ss715645257 | sc00003ln2130026_1727598_G_T_6052298 | 66.99 | 50,161,526 |
| ss715645256 | sc00003ln2130026_1706192_A_C_6030892 | 66.99 | 50,182,775 |
| ss715645254 | sc00003ln2130026_1685328_C_T_6010028 | 66.99 | 50,203,547 |
| ss715645252 | sc00003ln2130026_1665755_G_T_5990455 | 67.2 | 50,222,584 |
| ss715645251 | sc00003ln2130026_1591193_A_G_5915893 | 67.61 | 50,301,592 |
