# Supplementary material for: Fine mapping of an anthracnose-resistance locus in Andean common bean cultivar Amendoim Cavalo
Source: PLoS One. 2020 Oct 7;15(10):e0239763. doi: 10.1371/journal.pone.0239763 (PMC7540868; doi:10.1371/journal.pone.0239763)
Supplement: S2 Table — (DOC) [file pone.0239763.s002.doc]

**Table S2** Simple sequence repeats marker ID, motif, size, forward primer position, and forward primer sequences on version 1.0 of the reference genome of *Phaseolus vulgaris* and primer sequences

| SSR BARC ID | Motif | Size (bp) | Position Forward | Primer sequence |
| --- | --- | --- | --- | --- |
| BARCPVSSR01282 | (TA)15 | 30 | 48,640,985 | F- GCACTTCCCCTTGAATTGTG |
|  |  |  |  | R- TCCGATGATTTTAACCCTATGC |
| BARCPVSSR01309 | (AT)11 | 22 | 49,292,574 | F- GGTGGTCAACCCATACTGCT |
|  |  |  |  | R- GACATCTCAAATCCAAAACAAGTG |
| BARCPVSSR01313 | (AAT)8 | 24 | 49,368,487 | F- TCGGGATCCTTATCCAGTCTT |
|  |  |  |  | R- GCGTGCGTTAATTAATCATTTTC |
| BARCPVSSR01316 | (TCAC)11 | 44 | 49,449,846 | F- GCCACACACATACCATCTGC |
|  |  |  |  | GCCACACACATACCATCTGC |
| BARCPVSSR01342 | (AT)22 | 44 | 50,038,240 | F- CTTGAGGTGTCAGCCTAAATGA |
|  |  |  |  | R- CCCCCAATGGATTAAGGAAA |
| BARCPVSSR01377 | (TTA)10 | 30 | 50,856,104 | F- TTTTTCCTCTGATGTTGGCA |
|  |  |  |  | R- TTTCCCATTTTACCAACCAAA |
