# Supplementary material for: Fine mapping of an anthracnose-resistance locus in Andean common bean cultivar Amendoim Cavalo
Source: PLoS One. 2020 Oct 7;15(10):e0239763. doi: 10.1371/journal.pone.0239763 (PMC7540868; doi:10.1371/journal.pone.0239763)
Supplement: S3 Table — (DOC) [file pone.0239763.s003.doc]

**Table S3** KASP markers used to map the *Co-AC* resistance locus on the Andean common bean landrace Amendoim Cavalo

| ID | Position |  | Primer sequence | |
| --- | --- | --- | --- | --- |
| SS46 | 50,042,771 | F1 | | GAAGGTGACCAAGTTCATGCTCAATTTCTTACTTCATATTTTGTTCCCT |
|  |  | F2 | | GAAGGTCGGAGTCAACGGATTCAATTTCTTACTTCATATTTTGTTCCCC |
|  |  | R | | CTCCTGTCTCCTGTTTTATCCTGTCTA |
| SS47 | 50,065,488 | F1 | | GAAGGTGACCAAGTTCATGCTGACGTGGTATCCAGCGATCTC |
|  |  | F2 | | GAAGGTCGGAGTCAACGGATTGTGACGTGGTATCCAGCGATCTT |
|  |  | R | | AACGCCTTCACACTTTTGTTCAGTAGTT |
| SS48 | 50,093,966 | F1 | | GAAGGTGACCAAGTTCATGCTGAGCTTTTATTAGTAATTTGGTTTTGATTACT |
|  |  | F2 | | GAAGGTCGGAGTCAACGGATTGAGCTTTTATTAGTAATTTGGTTTTGATTACC |
|  |  | R | | GAGAGGAGTTACAAATTGCTAGTATGCAA |
| SS49 | 50,099,818 | F1 | | GAAGGTGACCAAGTTCATGCTCCAAAGCCACCTTTATATATAGAACCAAT |
|  |  | F2 | | GAAGGTCGGAGTCAACGGATTCAAAGCCACCTTTATATATAGAACCAAC |
|  |  | R | | CGATCATAACGGATAGAAAACCAGGTTTA |
| SS50 | 50,155,987 | F1 | | GAAGGTGACCAAGTTCATGCTAAAACCAGGTTTATTGTCCTAGTTTACAA |
|  |  | F2 | | GAAGGTCGGAGTCAACGGATTAACCAGGTTTATTGTCCTAGTTTACAG |
|  |  | R | | GTATCTCATCTTGTTGCAAGAGTGAATATA |
| SS51 | 50,161,526 | F1 | | GAAGGTGACCAAGTTCATGCTGGATTGAAAATGTTATACCCAGAAAAGC |
|  |  | F2 | | GAAGGTCGGAGTCAACGGATTGGGATTGAAAATGTTATACCCAGAAAAGA |
|  |  | R | | GTTCCATCTTTATCCATTCCCCTCTTTT |
| SS53 | 50,222,584 | F1 | | GAAGGTGACCAAGTTCATGCTATTTATGTGCTTTTCTGCTTCGTTCTG |
|  |  | F2 | | GAAGGTCGGAGTCAACGGATTATAATTTATGTGCTTTTCTGCTTCGTTCTT |
|  |  | R | | TGGTTTTTCGCACTATTATATCCACCAAAT |
| SS54 | 49,657,760 | F1 | | GAAGGTGACCAAGTTCATGCTCATGCCCATTTGAAGATGAACTGTTG |
|  |  | F2 | | GAAGGTCGGAGTCAACGGATTACATGCCCATTTGAAGATGAACTGTTA |
|  |  | R | | GAAGCCTAGAGGAACTAAGAAGCCTA |
| SS55 | 49,793,139 | F1 | | GAAGGTGACCAAGTTCATGCTCAATTATAAGTATCCACCATGAGAGTGT |
|  |  | F2 | | GAAGGTCGGAGTCAACGGATTAATTATAAGTATCCACCATGAGAGTGC |
|  |  | R | | GATAATCTGACCTCACCTGATTTTCTCTT |
| SS56 | 49,895,862 | F1 | | GAAGGTGACCAAGTTCATGCTATAAAACCTCTTGGTTCTGCAAGCC |
|  |  | F2 | | GAAGGTCGGAGTCAACGGATTCATAAAACCTCTTGGTTCTGCAAGCT |
|  |  | R | | TCCTTGCTTAGTTCATTGAATGTGGAGTT |
| SS57 | 50,301,592 | F1 | | GAAGGTGACCAAGTTCATGCTCATACACCAAGAGCCTCTCATCTTTT |
|  |  | F2 | | GAAGGTCGGAGTCAACGGATTATACACCAAGAGCCTCTCATCTTTC |
|  |  | R | | CCACAAGCACTTGGTTGGACTTGTT |
| SS92 | 50,527,176 | F1 | | GAAGGTGACCAAGTTCATGCTGCACTTGTTGTTAATTTTATCTTCTACAATTG |
|  |  | F2 | | GAAGGTCGGAGTCAACGGATTAGCACTTGTTGTTAATTTTATCTTCTACAATTA |
|  |  | R | | TTTGTTTTTTGGTTAATGCAGGGASCCTA |
| SS98 | 50,785,432 | F1 | | GAAGGTGACCAAGTTCATGCTGTTCTGGCAGAACAAATCCTGAAGA |
|  |  | F2 | | GAAGGTCGGAGTCAACGGATTGTTCTGGCAGAACAAATCCTGAAGT |
|  |  | R | | AGCRGAAGCTATTGCTCTTGCTGAAAT |
| SS102 | 50,377,247 | F1 | | GAAGGTGACCAAGTTCATGCTATTGTATACACTTGAGTTCATGGTCGT |
|  |  | F2 | | GAAGGTCGGAGTCAACGGATTGTATACACTTGAGTTCATGGTCGC |
|  |  | R | | CCTCCTTTCGGTATGCCACCCAT |
| SS95 | 50,442,472 | F1 | | GAAGGTGACCAAGTTCATGCTAATAAGAGAGCCCAGGTAGTGACAT |
|  |  | F2 | | GAAGGTCGGAGTCAACGGATTAAGAGAGCCCAGGTAGTGACAC |
|  |  | R | | GCCTAAAACAACCCAGATATGAGYACAT |
| SS165 | 50,386,692 | F1 | | GAAGGTGACCAAGTTCATGCTATGCATTATGAAAGATATGTGGCATGG |
|  |  | F2 | | GAAGGTCGGAGTCAACGGATTGAATGCATTATGAAAGATATGTGGCATGT |
|  |  | R | | TTTGAGATGAGGAAGTGGAAGTGGAATT |
| SS166 | 50,432,710 | F1 | | GAAGGTGACCAAGTTCATGCTTGGTACTCTGCAGATATCAGATAACA |
|  |  | F2 | | GAAGGTCGGAGTCAACGGATTGGTACTCTGCAGATATCAGATAACT |
|  |  | R | | CGACCACTTTATATTCTGCCTCTCAAAT |
