# Supplementary material for: Fine mapping of an anthracnose-resistance locus in Andean common bean cultivar Amendoim Cavalo
Source: PLoS One. 2020 Oct 7;15(10):e0239763. doi: 10.1371/journal.pone.0239763 (PMC7540868; doi:10.1371/journal.pone.0239763)
Supplement: S4 Table — The phenotype was obtained from the reaction of the 77 F3 plants to race 3481 of Colletotrichum lindemuthianum. Genotyping was achieved using the flanking KASP markers SS56 and SS92 (highlighted in orange) and the nine other KASP markers (highlighted in yellow) that enabled the positioning of the Co-AC locus in a 9,445 bp genomic region flanked by markers SS102 and SS165. A total of 54 out 77 F3 recombinant plants were assigned a recombination type from 1 to 16. AA = homozygous susceptible as PI 207262, BB = homozygous resistant, as the Amendoim Cavalo locus, AB = heterozygous resistant. (DOC) [file pone.0239763.s004.doc]

Table S4. Genotype and phenotype of 77 F_3_ recombinant plants used for fine mapping of the Co-AC anthracnose resistance locus. The phenotype was obtained from the reaction of the 77 F_3_ plants to race 3481 of *Colletotrichum lindemuthianum*. Genotyping was achieved using the flanking KASP markers SS56 and SS92 (highlighted in orange) and the nine other KASP markers (highlighted in yellow) that enabled the positioning of the *Co-AC* locus in a 9,445 bp genomic region flanked by markers SS102 and SS165. A total of 54 out 77 F_3_ recombinant plants were assigned a recombination type from 1 to 16. AA= homozygous susceptible as PI 207262, BB= homozygous resistant, as the Amendoim Cavalo locus, AB= heterozygous resistant.
